# The Impact of the COVID-19 Pandemic on Primary Emotional Systems and Emotional Regulation

**DOI:** 10.3390/ijerph18115742

**Published:** 2021-05-27

**Authors:** Rachele Mariani, Alessia Renzi, Cinzia Di Monte, Elena Petrovska, Michela Di Trani

**Affiliations:** 1 Department of Dynamic and Clinical Psychology, and Health Studies Sapienza, University of Rome, 00185 Rome, Italy; alessia.renzi@uniroma1.it (A.R.); cinzia.dimonte@uniroma1.it (C.D.M.); michela.ditrani@uniroma1.it (M.D.T.); 2 Derner School of Psychology, Adelphi University, Garden City, NY 11530-0701, USA; petre813@newschool.edu

**Keywords:** coronavirus pandemic, emotion regulation, primary emotional systems, mental and physical health

## Abstract

(1) Background: The COronaVIrus Disease 2019 (COVID-19) pandemic poses a unique challenge as a severe global crisis affecting physical and psychological health. The main purpose of this work is to study the impact of a traumatic event while also observing the human ability to adapt. One of the first theories to study the adaptive importance of the evolutionary lineage of the affective systems is referred to as BrainMind (Panksepp, 2010). This study aims to explore whether primary emotional systems (PES) and emotion regulation (ER) strategies show differences between the pre- and post- lockdown period; and if positive systems and specific emotion regulation pre-pandemic act as a protective or risk factor for mental and physical wellbeing. (2) Methods: 98 participants who had participated in a previous study before the pandemic were re-contacted to refill the Affective Neuroscience Personality Scale (ANPS) and the Emotion Regulation Questionnaire (ERQ) after the outbreak of the pandemic with the addition of the Symptom Checklist-90-Revised (SCL-90R). (3) Results: The results showed that the COVID-19 lockdown rules had an impact on Emotional Regulation and on a re-balancing of PES. Moreover, pre-pandemic expressive–suppressive ERQ strategies and ANPS SADNESS scores appeared as relevant risk factors, which predicted higher Global Severity Index (GSI) scores during lockdown. (4) Conclusions: The lockdown appears to have activated PLAY and CARE as protective systems, but has detuned the ability to positively reinterpret the situation.

## 1. Introduction

The Coronavirus-19 Pandemic posed a significant global challenge and particularly to Italy, as the first European country impacted by it [1]. This severe universal crisis has disrupted various crucial aspects of life and affected both the physical and psychological health of individuals facing this collective trauma [2]. COVID-19 has been defined as a cultural trauma, which in fact shared many of the characteristics that circumscribe this, including: a fundamental disruption of what is taken for granted in daily life; a potential loss of trust in leaders and social institutions; negative attribution of the media; and a contentious struggle with meaning to determine what happened and who is responsible. People have experienced the pandemic as traumatic, characterized by a loss of existential security, a biopolitical condition that can potentially create new modalities of subjection and subjectivation, shaping both collective and individual subjectivities [3]. Cultural traumas imply anxiety and suffering, but also opportunity. The latter stems from the human capacity to learn and adjust to new conditions; to reevaluate the world, as well as to live in it. COVID-19 was a real threat to human survival and the Italian government adopted isolation and social distancing as its first, and perhaps, most effective response strategy. These elements have, in turn, severely tested the stamina of individuals and contributed to a notable increase in psychopathology as a reaction to the pandemic [1,4,5,6,7,8].

In this context, it is of particular interest to focus on the emotional parts of personality by referring to Affective Neuroscience Theory [9,10], which is one of the most well-known theories in the emotional sciences. Panksepp was the first to coin the term, “Affective Neuroscience” [11] and posited that human personality refers to stable individual differences in emotionality, motivation, and cognition, resulting in behavioral action patterns. Furthermore, he stated that emotions are the oldest evolutionary parts of human personality which drive human personality traits and behavior. Several researchers have explained [12,13,14] the role of emotions in relation to personality and how they influence human relationships. Panksepp et al. looked at the brain structures that underpin human emotions using neurobiology, ethology, and evolutionary results. At the heart of human emotional processes are three positive and three negative emotional structures (capitalizations denote advanced scientific jargon) which proposed that six primary emotional systems (PES) have been equivalently conserved across the mammalian brain. These phylogenetically old systems function as tools for survival and endow mammalian species with important brain systems to successfully interact with the environment. According to Panksepp, the primary positive emotions are: SEEKING, CARE, and PLAY; whereas the primary negative ones are: FEAR, SADNESS and ANGER [15,16]. These systems reflect embedded tools for survival which are highly evolutionary; imbalances in these different systems are associated with psychopathological characteristics [17]. For example, higher FEAR/SADNESS, along with lower SEEKING levels, represent the state of depression [18].

Following this notion, emotional regulation is strictly related to the internal primary emotional system (PES) which individuals have built during their own life.

PES influences emotion regulation strategies and structures specific relationship patterns between self and others and between self and the environment. Therefore, each person, throughout the course of their lifetime, tends to establish emotional strategies that balance the basic emotional systems. A traumatic event or a completely new situation will involve a need for each person to readjust their positive and negative systems in order to ensure survival [15]. For instance, the ability to feel the support of others during social distancing is a subjective ability connected to PES [10]. Psychological health is based on the development of harmonious and balanced positive and negative emotional systems. This balance influences higher mental processes and, on the other hand, conflict or imbalance can generate psychological suffering. The pandemic is a real attack on humanity. To protect the population, the Italian government quickly instituted social distancing rules and instituted a lockdown for all the population, except for health workers involved in protecting the health of citizens.

Utilizing the framework of Affective Neuroscience, PES should contribute to the rebalancing of emotional systems in order to adapt to the imposed rules and to survive through the usage of them. Our study emphasizes how people were triggered to generate emotional and behavioral strategies in response to the fear of extinction evoked by the virus and the collective trauma surrounding it. Panksepp coined the term “BrainMind,” which intentionally conflates ‘brain’ and ‘mind’ to reflect the importance of primary emotion in the influencing of attitudes, traits, and emotional strategies. We hypothesize that emotional BrainMind may detect adaptive strategies as a phylogenetically refined affective function over the course of human evolution. Furthermore, we hypothesize that diversified affective capacities can help reduce the stressful impact or, on the contrary, increase its effects. More specifically, we hypothesize that:

(1) PES and emotion regulation strategies will show differences between the pre-lockdown and lockdown period, thus highlighting possible changes in ways of coping with the critical experience of the pandemic;
(2) Both pre-pandemic PES and ER strategies will predict individuals’ mental and physical wellbeing (or psychopathology symptoms) during the lockdown period, acting as protective or risk factors in dealing with this traumatic situation.

## 2. Materials and Methods

### 2.1. Participants

A total of 98 healthy participants (46 males, 52 females) took part in this study. All participants were between the ages of 18 and 70 (M = 39.3; SD = 16.6); had an adequate understanding of the Italian language and were living in Italy at the time of the lockdown; were subject to lockdown social restriction rules; and possessed the technical ability to access the online platform to complete questionnaires. Our sample was extracted from a previous database of people who had participated in a personality and PES study prior to the pandemic. We therefore excluded people who had reported psychiatric diagnoses; those who reported taking medication for psychiatric reasons; and individuals who were working as healthcare professionals during the pandemic. Healthcare professionals were in fact the only professionals excluded from the lockdown and the rule imposed by the Italian government through the use of the slogan, “stay at home”.

### 2.2. Procedure

The participants involved in the study had taken part in a previous study about three months before the pandemic, providing the availability to be contacted for future studies. About 200 people were invited to respond to a new online questionnaire during the lockdown and 98 people answered the new questionnaire. The first administration of the test protocol took place about three months before the outbreak of the pandemic, between November and December 2019. For the first assessment the participants were enrolled using snowball sampling, and subjects were invited to participate in a study of personality and emotion regulation strategies. The surveys were made available through an online platform where participants gave their informed consent before completing the self-administered questionnaire and indicating their willingness to be contacted for a follow-up. For the second assessment during imposed lockdown in Italy (in April 2020) the participants were invited to repeat the compilation of the questionnaire. However, considering the difficult period the world was facing, in the second compilation there was an additional questionnaire which assessed mental and physical symptomatology and asked participants to share how they felt during the global health emergency lockdown. The survey protocol received ethical approval from the Department Ethics Committee.

### 2.3. Measures

A socio-demographic questionnaire was designed to collect information concerning age, gender, education level, relationship/social status, and current or previous clinical and mental diagnoses.

The Symptom Checklist-90-Revised (SCL-90-R) [19] is a 90-item self-report questionnaire that measures mental and physical symptoms which have occurred in the previous week. Each item is rated by respondents on a five-point Likert scale (0–4) ranging from having caused no discomfort to having caused extreme discomfort during the past week. The SCL-90-R has nine subscales, and the Global Severity Index (GSI) score reflects overall mental and physical distress. The questionnaire showed adequate test-retest reliability, internal consistency, and concurrent and discriminant validity [19]. In the present study, Cronbach’s alpha of the total scale was α = 0.93, whereas clinical subscales ranged from α = 0.72 to α = 0.87.

The Affective Neuroscience Personality Scale, version 2.4 (ANPS) [20] is a 112-item self-report questionnaire based on Panksepp’s studies and was derived from the description of PES. The items are based on a four-point Likert scale. Six subscales, representing PES, were identified: SEEKING, CARE, PLAY, FEAR, ANGER, and SADNESS/PANIC. The questionnaire showed satisfactory internal consistency and adequate concurrent and discriminant validity [20]. In the present study, Cronbach’s alpha values for the subscales were PLAY: α = 0.78; SEEK: α = 0.72; CARE: α = 0.71; FEAR: α = 0.81; ANGER: α = 0.78; SADNESS/PANIC: α = 0.74).

The Emotion Regulation Questionnaire (ERQ) [21] is a 10-item self-report scale designed to measure the tendency of respondents to regulate their emotions in two ways: Cognitive-Reappraisal (CR) and Expressive-Suppression (ES), which represent the subscales. Respondents answer each item on a seven-point Likert scale (1-7). The questionnaire showed good internal consistency [21]. Cronbach’s alpha values for the present study were: CR: α = 0.82 and ES: α = 0.73.

### 2.4. Statistical Analysis

All statistical analyses were performed using the Statistical Package for Social Science version 25 (SPSS version 25). Data is reported as means and standard deviations for continuous variables and as percentages for discrete variables. The SCL-90 and demographic variables were analyzed using Pearson’s correlation analysis. The t-test for paired samples was applied to explore differences between the pre-lockdown (Time1) and lockdown period (Time2) in the affective dimensions investigated (ERQ and ANPS). Cohen’s d was computed in order to obtain standardized effect sizes. A multiple linear regression was performed in order to investigate the predictive effect of age, gender and pre-pandemic ANPS and ERQ scores on mental and physical symptomatology (GSI) evaluated during the pandemic. All the variables were entered simultaneously and were statistically significant (*p* < 0.05). Collinearity was tested (Tolerance and Variance Inflection Factor) assuming values were in the correct/accepted ranges.

## 3. Results

### 3.1. Descriptive Analysis

The participants had a mean age of 39.3 (SD = 16.6). Years of education averaged 15.5 (SD = 2.4); 45.8% had a High School Diploma, 41.7% had a Master’s Degree, and 12.5% had a Bachelor’s Degree. In addition, 45.8% indicated that they were married/cohabiting; 25.2% were unmarried/not cohabiting and living independently (or with roommates); 8.3% were divorced; and 20.7% were single and living with their families of origin. Age is significantly correlated to pre-pandemic and post-pandemic assessment of FEAR (r = −0.559, *p* < 0.00), pre-pandemic CARE (r = −0.331, *p* < 0.01), and not significant correlated to SCL-90R and ERQ. The same mean of SCL-90R is 0.62 (SD = 0.45) and 16% of the sample overcame the GSI clinical cut-off. More specifically, more than 30% of participants displayed elevated symptoms of depression, anxiety and obsessive compulsiveness. Primary Emotional Systems, ERQ, and SCL90 did not show any significance difference when comparing the level of education, gender assigned at birth, or relationship status.

### 3.2. Comparison Pre- and Post-Pandemic

In regard to changes in affective systems and emotion regulation dimensions, data analysis showed an increase in the CARE (*p* = 0.001) and PLAY (*p* = 0.027) systems of ANSP and a decrease in the cognitive-reappraisal (*p* = 0.001) dimension of the ERQ (see Table 1).

### 3.3. Regression Analysis

We hypothesized that PES and ERQ evaluated prior to the pandemic could predict mental and physical symptoms during the COVID-19 lockdown. To test this hypothesis, we conducted a linear regression analysis using GSI (SCL-90R) scores as dependent variables and pre-pandemic ANPS and ERQ scores, age, and gender as independent variables.

The model explains 51% of the GSI scores (*R*^2^ = 0.51; adjusted *R*^2^ = 0.38; *F* = 3.85; *p* = 0.001), thus indicating an adequate fit of the model tested. The independent variables that showed a significant effect were: expressive-suppression ERQ (β = 0.45; *t* = 2.71; *p* < 0.001) and SADNESS ANPS (β = 0.71; *t* = 3.65; *p* < 0.001). Age and gender did not show any statistically significant results.

## 4. Discussion

Consistent with previous studies regarding the impact of the COVID-19 pandemic on psychological well-being [4,5,22,23], the presence of psychopathological symptoms was found in the sample we examined. More specifically, participants displayed higher symptoms of depression, anxiety and obsessive compulsiveness. Italy was one of the first countries to be significantly affected by COVID-19 and social isolation measures were immediately enforced there. Social distancing and government-imposed lockdowns have effectively kept many people in their homes. Italy is among the countries with higher death rates from COVID-19, and with a higher average age than the rest of the world, especially during the first stage of pandemic [24]. The high number of deaths that hit the country exposed all citizens to great stress and concern for their own safety. At that time, our survey focused on detecting the psychological and physical effects of the situation, as well as understanding the modalities of emotional regulation of those who were experiencing the lockdown. The opportunity to be able to contact those who had participated in one of our research projects before the outbreak of the pandemic allowed us to verify the balance/imbalance of the PES and of the cognitive-reappraisal and expression–suppression emotional regulation strategies investigated. Elderly people were the most affected by COVID-19, and, therefore, we investigated whether age had an incidence with the variables explored and with psychopathological symptoms in the lockdown phase. The results showed that seniority is negatively related to FEAR in both pre- and post- evaluation, and also negatively related to CARE systems in pre-pandemic evaluation, which is not a significant relationship to psychological symptoms. Therefore, although the elderly is the most affected population, age does not emerge as a risk factor for psychological stress. The relationship with fear as a primary emotional system and young age is in line with the data showing that young people have suffered greatly during the pandemic, accentuated by perhaps feeling more exposed [25,26].

The results showed that COVID-19 lockdown rules had an impact on Emotional Regulation and displayed a re-balancing effect on PES. Further, during the lockdown, there was a decrease of ERQ cognitive reappraisal. Cognitive-Reappraisal (CR) generally has a buffer function which aids in the prevention of psychopathology [27]. CR is a flexible and adaptive function of emotion regulation, but the results displayed a decrease in it due to the traumatic impact of COVID-19. However, in regard to PES, we found an increase in the PLAY and CARE systems, which served as protective elements against danger. Physical PLAY is the most complex basic social emotion and persists after neo decortication [11]; CARE, or the maternal nurturance system, includes nurturance and social bonding and suggests that there is an intimate evolutionary relationship to maternal motivations. These results are consistent to studies which have shown that lower scores in the PLAY and CARE systems are linked to depression [28]. In analyzing ERQ and PES results, it could be said that the decrease in CR showed a reduction in the ability to regulate the positive reinterpretation of situations [29,30]. In other words, the results showed how people’s emotions manifest during traumatic events and how this, in turn, activated a rebalancing of their positive systems in attempt to cope with the stressful situation.

The second hypothesis tested whether specific PES and ERQ factors evaluated before the pandemic predicted psychopathological symptoms. A regression model showed that pre-pandemic variables, such as SADNESS and Expressive–Suppression were a risk factor and predicted higher psychopathological symptoms of GSI during lockdown. These results are consistent with recent results obtained during the COVID-19 pandemic, which showed the protective function of the ability to cope with anxiety and stress through cognitive reappraisal. On the other hand, expressive suppression served as a risk factor for the development of psychopathological symptoms [31,32]. This result is consistent with previous studies which found that other traumatic events, such as war or abuse, suggested that PES involved SADNESS [33,34]. Several developmental studies have highlighted how the persistence of specific negative emotions in children can predict future psychopathology. It was found that higher levels of sadness in adolescents predicted internalizing symptomatology [35]. Our results confirm a significant link between the dominance of a negative primary emotional system and psychopathological manifestation.

All of these findings need to be interpreted in the light of some limitations. First, the sample size of this study is relatively small. Furthermore, the sample was composed of volunteers available to be re-selected from a previous study, which may have introduced a selection bias. A further limitation is linked to having investigated only two macro strategies for regulating emotions, instead of explaining more specific modalities that are useful in the management of psychopathology [36].

## 5. Conclusions

In conclusion, as proposed by Cole et al. (1994) [37], the present findings align with their assumption that psychopathology is related to an imbalance of emotional experiences (positive and negative) within an individual. The prevalence and persistence of predominantly negative feelings, without the presence of positive emotions to balance the emotional baseline, can induce a greater risk of psychopathology when facing future stressful events. The imbalanced evaluation of the environment therefore increases the perception of risk and the negative evaluations of the strength of the individual. Our results also highlight that under the stress of the pandemic, the changing emotional factors are positive, which seems to indicate that individual balancing can be achieved through positive affect, while negative emotions appear to be less flexible.

This aspect can lead to future insights confirming the incidence of the role that negative experiences during the early stages of life, as well as dysfunctional emotional regulation models learned from childhood, can impact an individual’s mental health [38].

Our findings suggest that psychological interventions focused on the prevention of the imbalance of negative emotional systems can aid others in coping with negative feelings and therefore, in regulating and reinforcing resilience to stress [39].

The PES model suggests that the human adaptive capacity is able to cope with significant criticalities imposed by the environment and that flexible emotional systems are able help individuals adapt. It is recommended that all this be taken into consideration when developing the promotion of health in response to collective trauma through the reinforcement of flexible emotional regulation and the restoration of emotional balance. Our results also suggest that greater flexibility in re-establishing a good balance should be oriented towards the development of positive emotions and that the support for emotional regulation strategies should be aimed at the reappraisal of the situation.

## Figures and Tables

**Table 1:** *t* test comparison pre- and post-pandemic in ERQ and ANPS dimensions.

	Pre-Pandemic		Lockdown Period		T	P	Cohen’s *d*
	Time 1		Time 2				
	N = 98		N = 98				
ERQ	**Mean**	**SD**	**Mean**	**SD**			
Cognitive-Reappraisal	29.52	7.89	24.46	5.68	5.284	0.001 *	0.76
Expressive-Suppression	12.35	4.65	12.06	4.85	0.567	0.574	0.09
ANPS	**Mean**	**SD**	**Mean**	**SD**			
SEEK	30.17	4.45	29.80	5.03	0.698	0.489	0.10
FEAR	24.71	6.82	24.04	7.43	1.147	0.257	0.16
CARE	29.46	5.44	32.05	4.62	−4.245	0.001 *	0.61
ANGER	17.71	7.40	17.00	6.63	1.171	0.248	0.17
PLAY	25.40	5.98	26.60	5.44	−2.290	0.027 *	0.33
SADNESS/PANIC	22.77	5.26	22.81	6.11	−0.073	0.942	0.05

*t* test for repeated measure, * *p* < 0.05 gf (97); ERQ = Emotion Regulation Questionnaire; ANPS = Affective Neurosciences Personality Scales; SD = Standard Deviation.

## Data Availability

Data available on request due to restrictions privacy.
